# The Current Situation of Pea Protein and Its Application in the Food Industry

**DOI:** 10.3390/molecules27165354

**Published:** 2022-08-22

**Authors:** Parvathy Shanthakumar, Joanna Klepacka, Aarti Bains, Prince Chawla, Sanju Bala Dhull, Agnieszka Najda

**Affiliations:** 1 Department of Food Technology and Nutrition, Lovely Professional University, Phagwara 144411, Punjab, India; 2 Department of Commodity Science and Food Analysis, Faculty of Food Science, University of Warmia and Mazury in Olsztyn, Oczapowskiego 2, 10719 Olsztyn, Poland; 3 Department of Microbiology, Lovely Professional University, Phagwara 144411, Punjab, India; 4 Department of Food Science and Technology, Chaudhary Devi Lal University, Sirsa 125055, Haryana, India; 5 Department of Vegetable and Herbal Crops, University of Life Science in Lublin, Doświadczalna Street 51A, 20280 Lublin, Poland

**Keywords:** pea protein, functional properties, extraction, pea protein products

## Abstract

Pea (*Pisum sativum*) is an important source of nutritional components and is rich in protein, starch, and fiber. Pea protein is considered a high-quality protein and a functional ingredient in the global industry due to its low allergenicity, high protein content, availability, affordability, and deriving from a sustainable crop. Moreover, pea protein has excellent functional properties such as solubility, water, and oil holding capacity, emulsion ability, gelation, and viscosity. Therefore, these functional properties make pea protein a promising ingredient in the food industry. Furthermore, several extraction techniques are used to obtain pea protein isolate and concentrate, including dry fractionation, wet fractionation, salt extraction, and mild fractionation methods. Dry fractionation is chemical-free, has no loss of native functionality, no water use, and is cost-effective, but the protein purity is comparatively low compared to wet extraction. Pea protein can be used as a food emulsifier, encapsulating material, a biodegradable natural polymer, and also in cereals, bakery, dairy, and meat products. Therefore, in this review, we detail the key properties related to extraction techniques, chemistry, and structure, functional properties, and modification techniques, along with their suitable application and health attributes.

## 1. Introduction

Proteins are important biomolecules that play major roles in maintaining human health, and due to their vital functional properties, they are key ingredients in many food systems [1]. Over the past few years, plant-derived proteins have gained prime importance due to their higher ethical profile, increasing concern from animal welfare organizations for meat proteins, and increased exposure to animal-based protein greenhouse emissions [2]. The use of plant proteins is considered essential when animal-derived proteins fail to satisfy the requirements of the global population. The quality of the protein is mainly assessed by the essential amino acid profile as it fulfils the human needs and the protein’s ability to be digested and absorbed [3]. Animal proteins are considered complete proteins as they contain all essential amino acids. In contrast, plant proteins are considered incomplete as they lack some essential amino acids (lysine, threonine, and sulfur-containing amino acids such as cysteine and methionine) that humans need for proper growth. However, in some exceptional cases, plant proteins, including soybeans, pea, and grains (quinoa and amaranth), contain all the essential amino acids [4]. In addition, consuming plant-derived proteins lowers the risk of several metabolic diseases, such as diabetes, cancer, and heart-related disorders [2].

Pea (*Pisum sativum*) is the second most important crop in the Fabaceae family as they contain major components including protein (20–25%), fat (1.5–2.0%), carbohydrates in the form of starch (24–49%) and total dietary fiber (60–65%) including 10–15% insoluble fiber and 2–9% soluble fiber. They also contribute to non-starch carbohydrates, including sucrose, oligosaccharides, and cellulose. The minor constituents present are vitamins, minerals, phytic acid, saponins, polyphenols, and oxalates [5,6,7,8]. The most prominent mineral element present in pea is potassium (1.04%) contained in the dry and dehulled weight of peas, followed by phosphorous (0.39%), magnesium (0.10%), and calcium (0.08%), respectively. Moreover, they are also a good source of water-soluble vitamins, particularly rich in B-group vitamins [9,10]. The essential amino acids with a high lysine and threonine content are also present. However, it is deficient in sulfur-containing amino acids, including methionine and cysteine [11].

Pea protein is a relatively new kind of plant protein; thus, due to its sufficient availability, cost-effectiveness, nutritional value, and significant health benefits, it is more popular in the food industry [12]. The regular intake of food rich in pea protein helps to reduce the risk of cardiovascular diseases and diabetes. It may have protective effects against numerous cancers (breast, renal, and colon) [13]. Pea proteins are generally hypoallergenic and possess health benefits such as anti-oxidant, anti-hypertensive, anti-inflammatory, modulating industrial bacteria activities, and lowering cholesterol [14,15]. It is also a good source of bioactive small peptides, which can provide antioxidant activity with inhibitory activity towards angiotensin I-converting enzyme (ACE) for beneficial health effects [16]. The appetite-suppressing effects of peas may be related to high amounts of protein which may delay gastric emptying, attenuate glucose absorption and concentration and stimulate the release of appetite-regulating hormones [8].

The different applications of pea protein in food-related products include encapsulation for bioactive ingredients, extruded foods, and edible films. They are generally used as a substitution for cereal flours, fats, and animal proteins [17,18,19,20]. Pea protein’s functional properties and its fractions’ ability to form soft gels help develop and produce dairy analog drinks, fermented products, and curd. Solubility is an important property for functional ingredients in high moisture foods, such as emulsions, foams, and beverages [21]. Other properties like surface-active and structure-forming make them suitable for forming encapsulation and delivery systems [22]. As an industrial application, it can be used for pest control as an insecticidal protein proteinaceous cysteine proteinase inhibitor (CPI) found in pea [23]. Therefore, the main objective of the present work is to highlight the current status and industrial food application of pea protein. This review provides outlooks on the functional attributes of pea protein isolates, including protein structure, extraction methods, and physicochemical properties. The application of pea protein in food products and its effects on the health of the human body are also evaluated.

## 2. Extraction Process of Pea Protein from Pea

Pea protein is available in different forms, including pea flour, pea protein concentrates, and pea protein isolate. Mainly pea protein is utilized in the form of concentrate and can be produced through an acid hydrolysis process [24,25]. Pea protein extraction requires selecting appropriate treatments to maximize yields and estimate functional, nutritional, and structural properties that affect their applicability in the food industry [26,27]. In addition, several extraction techniques, including wet extraction, dry fractionation, salt extraction, micellization, and mild fractionation, are used to obtain pea protein concentrates and isolates [28]. Prior to protein extraction, pea seeds undergo pre-treatment steps such as cleaning, drying, sorting, dehulling, and splitting that allow the detachment of the hulls and the cotyledons from whole pulses; therefore, it facilitates protein extraction without affecting their techno-functional properties [29]. The extraction method, pH, duration of solubilization, number of washes, ionic strength, solvation ratio, temperature, extraction equipment, and filtration or purification techniques are some factors that influence the efficiency of the extraction as well as the protein isolate characteristics [30].

### 2.1. Wet Extraction: Alkali Extraction/Isoelectric Precipitation

Alkaline extraction/isoelectric precipitation is the most frequently used conventional technique for producing pea protein isolates (highly concentrated protein fractions) [31]. AE/IEP takes advantage of using high solubility of proteins under alkaline conditions. In contrast, minimal solubility appears at their isoelectric point (pI) around pH 4–5 and takes advantage of the same solubility properties for vicilin and legumin. Alkali-like NaOH and KOH are commonly used to maintain basic pH, breaking disulfide bonds in the protein, which enhances protein recovery and yield [32]. The yield of the protein is affected by some components like the size of the particle, the solvent used, pH of solubilization, extraction time, and water flour ratio [30]. A study by Boukid et al. [28] reported that the highest protein yield (80%) was obtained at pH (9.96) and water flour ratio at 15 *v*/*w*. The pea protein extraction at pH > 10 has been associated with severe starch swelling, resulting in starch contamination in the PP isolate/concentrate [12]. In addition, processes using alkaline pH > 10 or longer holding times can cause the isolated protein to tend to denature, thus reducing its functionality and solubility. The protein becomes highly charged, and the repulsion between the similarly charged amino acid residues of the protein increases at alkaline pH values. The native structure of proteins is disturbed, and the folded protein is destabilized and unfolded to minimize its free energy since the charge density of the folded protein is greater than that of the unfolded protein at extreme pH values. In addition to pH, temperature plays a vital role in stabilizing protein structure and folding since it also helps maintain covalent interactions within the protein structure [33,34].

**Figure 1:**
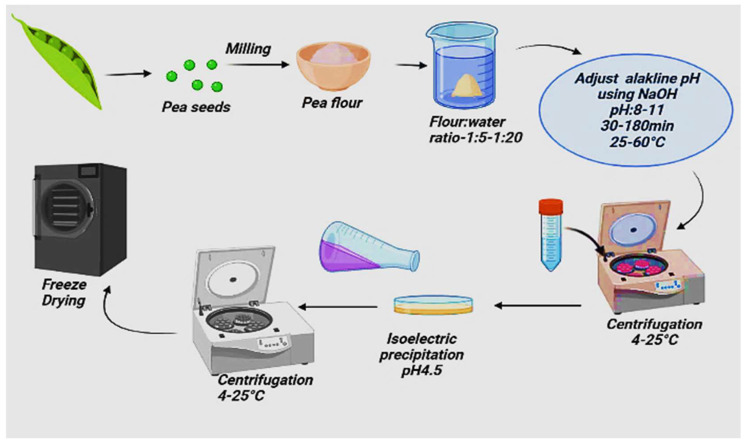
Extraction of pea protein by alkali extraction/isoelectric precipitation.

The defatted flour is dissolved in water in the alkaline extraction/isoelectric precipitation process (Figure 1). Then pH is adjusted to an alkaline range using NaOH or KOH, left for 30–180 min to maximize protein solubility. In the absence of the de-fatting process, protein-lipid interactions minimize the protein solubility and the yield of the isolate. The temperature is increased to 50–60 °C to facilitate solubilization, and high temperature tends to avoid limiting protein denaturation [35]. Afterward, the mixture is separated by centrifugation, then the supernatant is collected, and the isoelectric pH is adjusted. The precipitated protein is collected by centrifugation, then washed, neutralized, and dried by freeze-drying [28,36]. Therefore, the protein prepared by alkaline extraction has high digestibility and bioavailability. However, this method can produce a high yield while at the same time having some drawbacks. Alkaline conditions severely affect protein digestibility and cause adverse chemical reactions such as converting serine and cysteine residues to nephrotoxic lysinoalanine compounds, reducing protein bioavailability, and racemization of amino acids. This can be managed by maintaining a balance between alkali strength and protein extraction efficiency [12,34].

Another technique used to isolate protein from wet slurries is ultrafiltration (UF) with diafiltration. The protein extraction and starch removal processes are similar to those used in IEP, except that the extracted protein is passed through UF membranes [37]. One advantage is that small soluble compounds like oligosaccharides can be removed. Several advantages of UF over the IEF process include improved extraction efficiencies by changing the type of membrane used, molecular weight cut-offs, concentration and volume of the filtrate, and the addition of diafiltration to UF techniques [35,38]. A comparative study shows that the use of ultrafiltration results in the reduction of phytic acid content (28–68%), which helps to improve functional properties. Another study reported that alkaline extraction followed by isoelectric precipitation or UF produces high protein content compared to salt extraction due to higher carbohydrate solubility of salt solution. But one of the drawbacks of this method was not able to change the amino acid composition of PPI [39]

### 2.2. Dry Fractionation: Air Classification and Size Reduction

**Figure 2:**
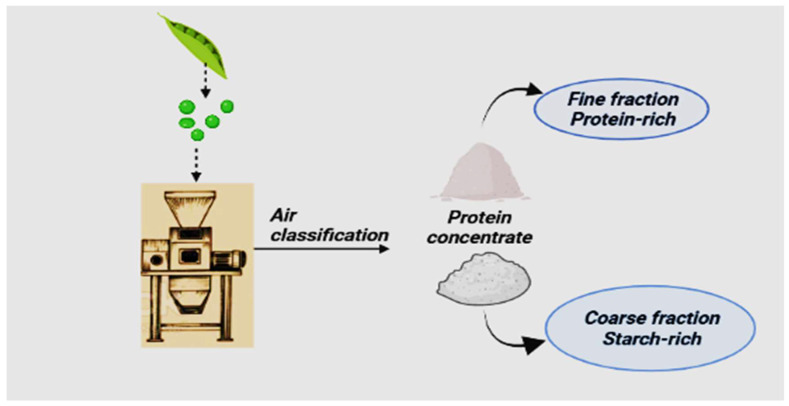
Extraction of pea protein concentrate by dry fractionation method.

Air classification and milling (size reduction) are commonly used to fractionate protein into thin or fine fractions (protein concentrate). In dry fractionation (Figure 2), dehulling of seeds is carried out as a pre-treatment because it reduces antinutritional factors (ANFs), separates bitter or astringent components, and improves color, resulting in a slight increase in the protein content [29]. The principle behind dry fractionation is the classification of flour into different particles size and the chemical composition after milling [40]. It depends upon the potentiality of milling to segregate protein bodies and other cellular components into different particle sizes [41]. Different milling technologies are used for this purpose, including roller, hammer, stone, pin, and jet milling. Among these, pin milling is used to reduce flour particles’ size [42,43].

Air classification of the pea flour (Figure 2) is performed in a spiral air stream into a fine fraction containing around 75% of the protein. After milling, some starch remains attached to the protein matrix, and some protein bodies still adhere to starch granules [37]. The separation of protein and starch can be improved by repeating air classification and milling methods. However, excessive milling can produce too fine particles, resulting in strong Van der Waals forces between particles and poor flow behavior. The fine fraction of pea protein concentrate is obtained with 50–77% [44]. Air classification is considered more sustainable than wet fractionation because it requires less energy and water and does not need a drying process or the addition of chemicals [45]. A limitation of this technique is that some fine and coarse particles may enter the wrong fraction due to random air turbulence and particle-particle collisions. This method is inefficient in separating particles that have similar particle sizes. Its major drawback is the low purity of protein concentrate compared to protein isolates [27].

In recent years, a tribo-electrostatic technique has been adopted for protein fractionation. In this process, particles are separated based on triboelectric charging properties induced by particle-wall and particle-particle interactions [46]. Protein gains a positive charge from these interactions, whereas fiber components gain a slightly negative charge; thus, they can be separated in an electrostatic field [47]. The combination of air classification and electrostatic separation obtained high purity protein concentrate. But more research is needed to improve and optimize this fractionation technique [48].

### 2.3. Salt Extraction and Micellization

**Figure 3:**
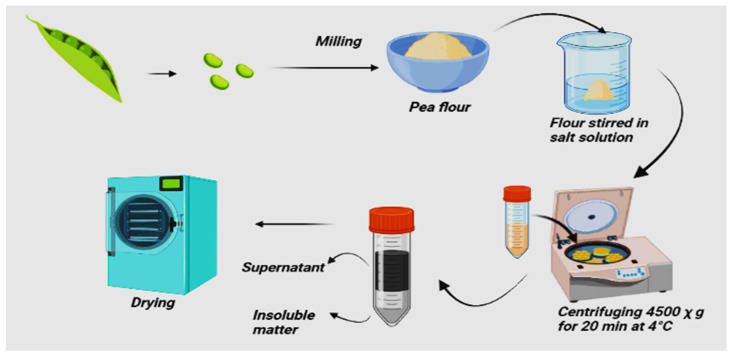
Schematic representation of salt extraction method of protein.

This method extracts proteins from seed material in salt solution at neutral pH (Figure 3). Salt extraction has the advantage of the salting-in and salting-out phenomena of proteins, followed by a desalting process that reduces the ionic strength of the protein environment [35]. Pea flour is stirred in a salt solution for 10–60 min with a specified ionic strength at a ratio of 1:10 (*w*/*v*). The insoluble matter is removed by settling, pouring, screening, and centrifuging. The supernatant is desalted and dried then the concentration and salt mixture are chosen based on the salting-in characteristics of the protein to be isolated as well as the salting-out characteristics of any unwanted proteins since proteins precipitate at an array of ionic strengths [11]. Other factors include adverse interactions between the salt and sample components and ensuring the use of food-grade salts [49]. Generally, salting-in of proteins appears at low ionic strength, between 0.1 and 1 M. Some benefits of SE are that extreme alkaline or acidic pH or elevated temperature are not required. The extraction takes place at the natural pH level of 5.5–6.5. Alkaline extraction tends to extract a slightly higher legumin content. In contrast, salt extraction is more favorable for extracting vicilin and convicilin due to the lower solubility of legumin in diluted salt solution than vicilin [39]. A study by Stone et al. [11] reported all extraction produces different solubilities in which SE produces high solubility (85.7–91.1%). This is because of the difference in surface characteristics of the protein due to the extraction method. The solubility is related to the folding of the protein and exposure of hydrophilic and hydrophobic groups, the latter leading to protein-protein interactions and insolubility. In general, SE isolates had better solubility, OHC, and foaming capacity than others, but it has low WHC. Although SE produces isolates with better extraction yield and functionality [12].

The salt extraction process, sometimes defined as micellization, is based on proteins’ salting-in and salting-out phenomenon. The micellization method induces precipitation of protein by adding cold water in the ratio of 1:3 to 1:10 (*v*/*v*) of high salt protein extract to water. Dilution of the protein solution forces solubilized proteins to regulate the low ionic strength through a series of dissociation reactions to form lower molecular aggregates [12]. When it reaches a critical protein concentration (CPC), the aggregates combine into a comparatively low molecular weight species called micelles, precipitated from solutions. To maximize micelle formation, the diluted solution is left to stand for some time [45]. Then, the precipitated protein can be recovered by centrifuging, washing, resuspending, and spray drying. The micellization process has the advantage of being a mild process with less extreme pH changes, resulting in reduced protein denaturation during the process [50]. A limitation of the micellization method is the low protein recovery due to a lack of protein solubilization [11].

### 2.4. Mild Fractionation

**Figure 4:**
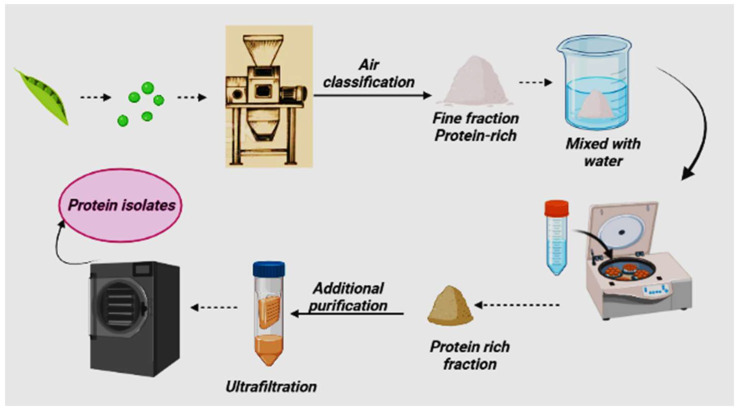
Extraction of pea protein by mild fractionation method.

The mild fractionation process (Figure 4) produces pea protein isolates using a hybrid approach method [51,52]. Hybrid methods mean adopting certain steps from both dry and wet methods. In this process, the fine fraction of pea flour is immersed in water and then fractionated by a layer-by-layer separation by using centrifugation forces or additional purification to increase its purity (70–90 g protein in 100 g dry matter) [28]. A combination of dry and wet processes can be used to develop efficient hybrid separation methods for producing protein ingredients with acceptable purity and functionality. At the same time, it requires only minimum effort for processing and costs [53].

The above extraction methods show that none of them can be considered to be best in all criteria. However, when comparing all these methods, alkaline extraction is electric precipitation that achieves a high yield, high solubility, and high native protein product. Accordingly, solubilization and precipitation steps significantly affect protein profile, solubility, conformation, and nativity [11,39] (Table 1).

## 3. Chemistry and Molecular Structure

Pea seed is rich in protein, carbohydrates, and some minerals, but the nutritional content of the seed depends on environmental and genetic factors [5]. Pea seeds usually contain 20–25% protein, 40–50% starch, and 10–20% fiber [8]. Pea protein can be classified into four major groups: albumin, globulin, prolamin, and glutelin, where the majority are globulins (65–80%) and albumins (10–20%) [12,59]. Globulins are the main storage proteins and are soluble in salt solutions. Based on the sedimentation coefficient, globulin has two main fractions, legumin (pI 5–6) and vicilin (pI 4–6), which belong to 11 S and 7 S seed storage protein classes, respectively, and a minor amount of a third type known as convicilin. Due to salt solubility, globulin can be degraded during seed germination to provide nutrients for plant growth [7,60]. Legumin is a hexameric protein with a compact quaternary structure that is stabilized by electrostatic, disulfide, and hydrophobic interactions having a molecular weight of 320–400 kDa with a beta-sheet-rich structure [61]. The mature proteins contain six subunits bound by noncovalent interactions, each of these subunit pairs has an acidic (40 kDa) and a basic (20 kDa) chain linked by a single disulfide bond. Disulfide bridges link the α-chain and β-chain of legumin, and the hydrophilic α-chains are on the molecule’s surface while hydrophobic sections are immersed in the inner surface to minimize contact with water. There is some heterogeneity in each α and β chain, but the α chain has leucine as the N-terminal amino group and is dominated by glutamic acid. In contrast, the β-chain contains more alanine, valine, and leucine and has glycine as the N-terminal amino group [25,62] (Table 2).

Vicilin is a sparsely glycosylated trimeric protein with a molecular mass of 150–170 kDa and a sedimentation coefficient of 7S. It is a heterogeneous combination of polypeptides, lacking cysteine residues, and cannot form disulfide bonds. Each monomer is 47–50 kDa and is composed of three subunits (α, β, and γ), and these subunits vary because of post-translation processing that results in fractions of 12–36 kDa [11,12,59,68]. Vicilin proteins have low concentrations of sulfur-containing amino acids (methionine, cysteine) and tryptophan and higher concentrations of basic (arginine, lysine) and acidic (aspartic acid, glutamic acid) amino acids [69]. These amino acids profile of pea protein (Table 2) have been identified as limited in pea, and increasing their concentrations has been identified as a major role in improving the nutrition of peas. Vicilin has formed amyloids in pea cotyledon cells that are protein aggregates with unique physicochemical properties resistant to protease action and vicilin amyloid resistant to gastrointestinal digestion [70]. The heterogeneity of vicilin is more complex than the heterogeneity of legumin. Its heterogeneity derives from a combination of factors, including the production of vicilin polypeptides from several small gene families encoding different primary sequences, proteolytic processing, and glycosylation [71]. Convicilin is the third storage protein with a molecular mass of 70 kDa, forming trimers of 210 kDa (or 290 kDa including an N-terminal extension) with three convicilin molecules or heteromeric trimers with vicilin [72]. This protein has a significantly different amino acid profile than both legumin and vicilin and is very low in carbohydrates [73]. They contain sulfur-containing amino acids and a highly charged N-terminal extension. They differ from vicilin in having a sulfur-containing amino acid, cysteine [25]. They have extensive homology with vicilin along with the core of its protein. Still, they are characterized by the presence of a highly charged hydrophilic N-terminal extension region consisting of 122 or 166 residues [51].

Albumin (2S) is a water-soluble protein having 18–25% of the total protein in pea seeds [62]. It is a metabolic and enzymatic protein showing cytosolic function, comprised of molecules that play functional roles in seedling growth [74]. It contains enzymes, protease inhibitors, amylase inhibitors, and lectins with molecular masses ranging from 5-80kDa [75]. Pea seeds distinguish two small MW albumins (PA1a and PA1b) in which PA1a has 53 amino acids with 6 kDa and PA1b has 37 amino acids with 4 kDa [76]. The comparison of gene sequences shows some similarities between PA1 and some low molecular weight proteins from the seeds of a wide range of monocot and dicot plants. The MW albumins PA1a and PA1b have exceptionally high levels of cysteine (7.5% and 16.2%, respectively), and PA1b has the ability to act as an insecticide in biological control [7]. The ratio of globulin and albumin in pea protein isolates (PPI) varies due to genetic variants and processing conditions, which affect the physicochemical properties of PPI [60,77]. The detailed classification of protein fraction and its molecular characteristics are given in (Table 3).

Another group of plant storage protein is prolamin, which presents a small amount in pea seeds and is characterized by high glutamine and proline contents, generally soluble only in strong alcohol solutions (70–80%), light acid, and alkaline solutions. Prolamin does not coagulate by heat but hydrolyzes to proline and ammonia [7]. Glutelin, an insoluble protein, a class of prolamin-like proteins found a minor amount in pea seeds and constitutes a major component of protein composite as gluten. Glutelin is soluble in dilute acids or bases, chaotropic or reducing agents, and surfactants and is rich in hydrophobic amino acids such as phenylalanine, valine, tyrosine, and proline [12].

## 4. Modification Techniques to Improve Functional Properties

Protein modification means the process of modifying the molecular structure or chemical groups of a protein by specific methods for better functional properties. Modifying their physicochemical properties and finding their limitations make plant-based proteins them multi-functional components for food systems. Generally, the protein modification methods are broadly categorized into physical, chemical, and biological [13,80].

### 4.1. Physical Modification

Improving protein functionality by the physical method is simple and is not based on chemical or enzymes. This method of protein modification has gained significant interest and also avoided the harmful consequences of chemical residuals.

#### 4.1.1. Heat Treatment

Conventional heating is one of the common techniques for the physical changes in structural and functional properties of plant-based protein [81]. Mild heat helps the protein unfold, resulting in an intermediate molten globule state with enhanced functional properties. However, the high-temperature treatment causes irreversible changes in the protein structures, including hydrophobic, disulfide, and electrostatic, resulting in lower solubility due to protein aggregation and precipitation [79]. Although heat treatment is not favorable for improving protein solubility, emulsions stabled by heat-treated PPI exhibited better creaming stability than those formed by unmodified PPI. Several studies reported that heat treatment enhanced the emulsion ability of protein at pH 7.0 but reduced its foaming properties [82,83].

#### 4.1.2. High-Pressure Treatment

High hydrostatic pressure (HHP) is a non-thermal process adopted from an isostatic pressing process where hydrostatic pressure in the range of 100–800 MPa is applied in all directions for several minutes. It is mainly introduced for the preservation of milk [84,85]. The applied pressure and holding time depend on the type of product treated. HHP has been used for specific functions, including enzyme and microbial inactivation, texture modifications, and emulsification. Generally, enzyme inactivation requires high pressure than the pressure used for microbial inactivation [86]. HHP process helps to improve the protein hydrophobicity whereas reduces the solubility due to its ability to expose buried sulfhydryl groups after protein unfolding, denaturation followed by aggregation, coagulation and improves its functional properties [87]. In terms of these structural changes, increased surface hydrophobicity, sulfhydryl, and secondary structural changes resulted in better thermal stability and emulsifying properties of the protein [88]. The foaming stability and emulsifying property of pea protein are significantly improved by high pressure (HP) supercritical CO2 treatment at certain pH levels, where its solubility is not significantly improved [82,89].

#### 4.1.3. Heat with Shear Treatment (Extrusion)

Extrusion means a grouping of mechanical shear, pressure, and heat in which the ingredients are consistently mixed and high mechanical stresses are generated by a large rotating screw under high temperature (90–200 °C) and pressure (1.5–30.0 MPa). Since it is a high-temperature short time (HTST) process, it has been used for a wide range of purposes, such as the destruction of microorganisms, enzymes, and naturally occurring harmful substances as well as used for gelatinization of starch [90]. Extrusion causes protein molecules to unfold, denaturation, and realign which not only improves their functional properties but also forms a texture [91,92]. In the case of pea protein, the molecular weight changes after the low-moisture extrusion process, and the secondary structural changes are stated by the identification of formed α-helices, β-sheet, non-covalently bonded β-turn, or anti-parallel β-sheet structures [93]. Extrusion at high temperatures and pressure harms the anti-nutrients and improves proteins’ digestibility by raising their amino acids’ availability. This process is also used as a pre-treatment for other protein modification methods such as hydrolysis and glycation by making the amino acids folded in the inner layer of the proteins [94].

#### 4.1.4. Cold Atmospheric Pressure Plasma Treatment

Cold atmospheric plasma processing is based on the application of cold plasma, the fourth state of matter, and is accomplished by merging heat, mechanical, nuclear, and electrical energy sources over a wide range of temperatures and pressures. CAPP is subdivided into thermal plasma and non-equilibrium plasma [86]. The benefit of this process include uniform treatment without any heat damage and the absence of required hazardous solvents. The main goal of this treatment has been to ensure microbial product safety and enzymatic stability [95]. CAPP’s high energy causes the breakage of chemical bonds or initiation of chemical changes. It is also used to modify the surfaces and biopolymers. Furthermore, it is used to enhance the functional properties of plant-based proteins as well as to reduce the size of the plant-based protein molecules and aggregates [96,97]. Plasma treatment helps to modify the secondary structure and physicochemical characteristics of plant-based proteins by increasing the content of disulfide bonds. Cold plasma induces unfolding and modification of the secondary structure of pea protein resulting in improved solubility, emulsifying ability, and water holding capacity [98]. In addition to this, it also improves the gelling properties of pea protein by allowing it to form gels when heated below 90 °C and also increasing PPI solubility [95,99].

#### 4.1.5. Ultrasonic Treatment

Ultrasonic (US) treatment is a non-thermal green technology that can modify protein conformation and structure due to the disruption of non-covalent bonds [39]. Thus, it can destroy the secondary structure and also partially denature the tertiary and quaternary structure of the protein without any changes in the primary structure [80]. US treatment significantly improved PPI solubility, and its solubility further increased with increasing sonication time [100]. Moreover, high-intensity US processing effectively improved PPI’s foaming properties and emulsifying activity [101].

### 4.2. Chemical Modification

Chemical modification has been widely used due to its ease of operation, efficiency, and low cost. The chemical modification of protein can be achieved by eliminating some components from protein structure and adding some functional moieties.

#### 4.2.1. Glycation

Glycation is a non-enzymatic glycosylation reaction to alter protein functionalities and does not require exogenous chemicals [102]. Glycation is inspired by naturally occurring covalent bonds between proteins and polysaccharides in some biopolymers, such as Arabic gum [103]. It is also achieved chemically by Millard reaction, or it can be obtained by linking some enzymes like transglutaminase. Chemical glycation occurs by the covalent conjugation method through controlled heating in the presence of water by different methods, including dry heating, wet heating, and molecular crowding [104]. Several factors should be controlled to improve the yield, quality, and functional properties of glycated proteins, including time, temperature, nature, pH, water activity, and concentration of reactions [105]. The nature and reactivity of the saccharides are important factors for improving the techno-functionality of plant-based proteins through the Maillard reaction [106]. The changes in the aromatic profile of glycated protein indicate that the Millard reaction not only diminishes beany flavor but also provides an application as ingredients in food formulations without any unfavorable effect on their organoleptic properties [102,107]. Since solubility is the biggest weakness of pea protein after glycation with gum arabic increases by 5.5% solubility [108].

#### 4.2.2. Acylation (Acetylation and Succinylation)

Acylation is the addition of an acyl group to a protein using acyl anhydrides and halides. Based on the acylating agent used (acetic or succinic anhydride) the process is classified into acetylation and succinylation [82]. Acetylation adds acetyl groups to protein amino groups in a covalent manner and is usually performed using acetic anhydride as a reagent, resulting in the unfolding of the protein (the electrostatic attraction is reduced). Hydrophilic groups are exposed, and therefore, hydrophilicity increases, improving solubility. Other functional properties of the protein also benefit from this. The succinylation is carried out by adding succinic anhydride; in the process, succinyl groups are incorporated into a protein. As a result of this reaction, an exchange of charge takes place from positive to negative in lysine residues [109]. Several studies reported succinylation is more effective in altering protein conformations and functional properties because eliminating lysine’s positive charges increases its negative charges. This phenomenon directly affects the repulsive electrostatic forces and causes more unfolding of the native form. This change reduces interactions between protein molecules as water molecules establish interactions with the unfolded protein and increase solubility [110]. Acylation alters the secondary structure and tertiary conformation of plant-based proteins, making them more hydrophobic with the possible improvement of their functional properties without adverse effects on their nutritional value [111]. The effect of succinylation on secondary structure helps to improve solubility, foaming properties, emulsion stability, and the water holding capacity of pea protein [112].

#### 4.2.3. Deamidation

**Figure 5:**
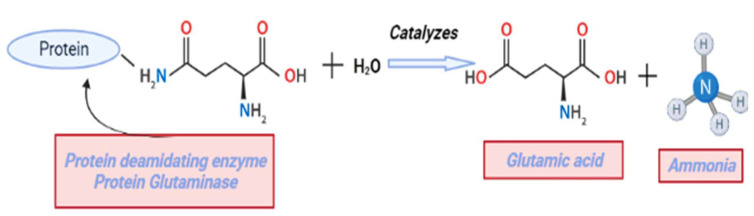
Schematic representation of deamidation process of pea protein by the deamidating enzyme.

Deamidation is the process of converting the amide groups of glutamine and asparagine residues into carboxyl groups by increasing the negative charge of the protein. In the case of acylation needs chemicals, but in deamidation process can be managed under mild conditions and without the use of any additional molecules. As a result, it is considered to be a safe method of protein modification in food systems [81,113]. Deamidation can be carried out by different methods such as alkali, acid, enzymatic and cation-exchange-resin treatment, in which the alkali and acid treatments being the most and least commonly used [114]. Glutaminase is the most widely used enzyme for the deamidation of plant-based proteins. Protein-glutaminase is a new type of protein-deamidating enzyme. It catalyzes the deamidation of glutamine residues in substrate proteins or polypeptides into glutamic acid and also releases ammonia shown in (Figure 5) [115]. A study by Fang et al. [116] observed that the glutaminase deamidation process improves solubility and techno-functional properties of pea protein and reduces the unpleasant beany flavor, bitterness, and lumpiness.

### 4.3. Biological Modification

In a biological modification, the most common protein modification methods are enzymatic modification and fermentation.

#### 4.3.1. Fermentation

Fermentation is the process used as a traditional and cost-effective biological method for the modification of plant-based proteins. Different starter cultures are used for fermenting plant proteins, such as lactic acid bacteria, yeast, mold, and bacillus strains, among which lactic acid bacteria are the most common [117]. Fermentation helps to improve protein solubility, water and oil holding capacity, and forming properties. Fermentation is used to improve the structure and functional properties and enhance nutritional properties [118]. It has improved the digestibility of pea protein by reducing the non-nutritive compounds that inhibit digestive enzymes and promoting protein crosslinking (i.e., tannins, trypsin, α-galactosides, and chymotrypsin inhibitors) [119]. Fermentation also improved mineral bioavailability, as microbial metabolism generates organic acids, which then form soluble complexes with mineral compounds preventing the formation of insoluble mineral-phytate complexes [68].

#### 4.3.2. Enzymatic Modification

**Figure 6:**
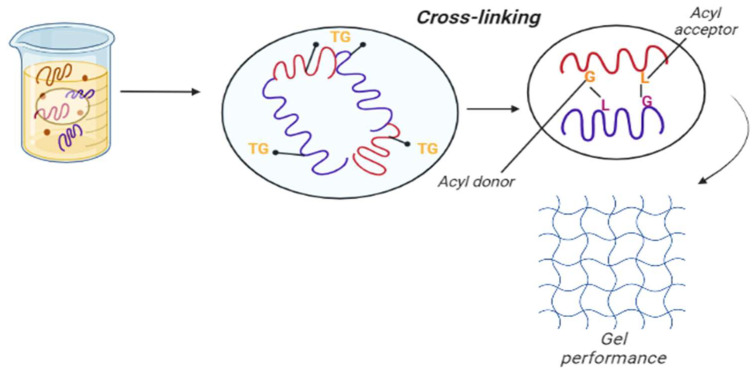
Enzymatic modification of pea proteins by a crosslinking method.

An enzymatically modified approach is considered to be cleaner and more efficient than physical and chemical modification (Figure 6 and Table 4). Another advantage of enzymatic modification over chemical approaches is enzymes’ fast reaction time and specificity [120]. This type of protein modification is classified into enzymatic hydrolysis and crosslinking methods. Enzymatic modification is an alternative method for improving the emulsification characteristics of pea protein by the breakdown of protein structure. Microbial transglutaminase (MTG) is an enzyme commonly used to cross-link protein. It is isolated from specific bacteria such as Streptoverticillium mobaraense; the latter can be achieved by the enzymatic formation of covalent bonds using transglutaminase and laccase by catalyzing acyl transfer reaction between a γ-carboxamide group of protein-bound glutamine and lysine, thereby affecting gel performance of protein [72]. During microbial transglutaminase treatment, pea protein’s albumin and globulin fractions show a different behavior, and the albumin fraction is not good for gelation. The changes in structure and conformation of proteins are responsible for improving techno-functionality [121].

Protein hydrolysis is a catalytic reaction between proteolytic enzymes and protein substrates that results in peptide bond cleavage and substrate splitting into short-chain peptides and amino acids with lower molecular weights [122]. The process typically reduces molecular weight, increases the number of ionizable groups, and exposes the hydrophobic group buried in the protein core, potentially improving the protein’s solubility, hydrophobicity, emulsifying, and foaming properties [123]. The type of enzyme, the nature of the protein substrate, the enzyme to substrate volume ratio, process conditions (pH, temperature, and pressure), and the availability or absence of proteolytic inhibitors are the factors that influence the enzymatic hydrolysis of proteins [124]. Many researchers use this structure-modifying technique to effectively improve the solubility of pea protein at different pH values.

## 5. Techno Functional Properties

The application of proteins in food products highly depends on their functional properties. The functional properties are classified based on the mechanism of action, including properties related to hydration (solubility, water, and oil holding capacity), protein structure and rheological characteristics (gelation and viscosity), and surface characteristics (foaming, emulsifying properties) [49]. The extraction techniques and cultivar variations play important roles in determining the functional properties of proteins [11]. In general, the functional properties of proteins are influenced by many factors that significantly impact the behavior of proteins in food processing, storage, and consumption [125]. Factors can be divided into two groups: intrinsic and extrinsic. Intrinsic factors are amino acid composition and sequence, shape, size, hydrophobic/hydrophilic ratio, conformation, and reactivity. Extrinsic factors affecting the techno-functional properties of pure proteins include pH, ionic strength, temperature, conformation, hydrophobic/hydrophilic ratio, and extraction process [61].

### 5.1. Solubility

Solubility is a property used to measure the degree to which one compound gets dissolved into another. Other definitions include the ratio of protein present in the liquid phase to the protein present in both the liquid and solid phases under thermodynamic equilibrium conditions [12]. Protein solubility depends on the hydrophilic or hydrophobic balance of the protein molecule but mainly on the molecular surface composition in terms of polar or non-polar amino acids, which in turn affect the thermodynamics of protein-protein and protein-solvent interactions [109]. The most commonly used solvent is water or buffer. Protein solubility is one of the most commonly measured techno-functional properties of food proteins and can affect other protein properties such as gelation, foaming, and emulsification [79]. Several factors can affect solubility, including pH, ionic strength, temperature, solvent type, and protein concentration [49]. The surface properties of proteins, particularly the amount and distribution of hydrophilic and hydrophobic amino acid residues on the surface, can affect a protein’s behavior in solution. In water, hydrophilic amino acid residues are more oriented towards the solvent interface. In contrast, most of the hydrophobic residues are buried in the inner of the protein to minimize free energy. The remaining hydrophobic residues on the protein surface create hydrophobic spots that inhibit solubility [12]. Extraction and dehydration play important roles in protein solubility by affecting protein surface hydrophobicity, exposing hydrophobic residues, and increasing hydrophobic interactions between proteins [74]. Commercial pea protein has a lower solubility in wet extraction due to heat-induced denaturation during spray drying [82]. Besides wet extraction, more innovative dehydration techniques have been proposed to preserve the native form of proteins and improve the solubility of pea protein [11,52,82]. Some alternative strategies to improve pea protein solubility are controlled enzymatic hydrolysis [126,127,128], the use of additives, or sonication treatments [73,129]. Another factor to consider is the isoelectric point (pI), pH values above and below the pI, and increased solubility due to electrostatic repulsion caused by net positive and negative charges on the protein surface [110]. A protein has the lowest solubility at its isoelectric pH because it carries zero net charges since the hydrophobic interactions between the protein molecules are larger than the electrostatic interactions between the protein and water molecules [130]. In these conditions, hydrophobic interactions between neighboring proteins can lead to aggregation, and precipitation occurs once the aggregates are adequate in size and number [12]. In general, the solubility of pea protein is strongly pH-dependent, with a minimum solubility between pH 4 and 6 (less than 30%) and a maximum solubility above pH 6 and below pH 4 (about 80%) [83,131].

### 5.2. Water Holding Capacity

The water holding capacity (WHC) of a protein is defined as the amount of water absorbed by 1 g of protein. It is a crucial functional property as it affects a product’s texture and flavor binding [132]. Water binding occurs through a combination of ion-dipole, dipole-dipole, dipole-induced dipole, and hydrophobic interactions. The relation between water and protein is influenced by the structure of the protein matrix, particularly the pore size [12]. Generally, bound water is tightly associated with proteins, meanwhile, retained or immobilized water becomes trapped in the protein matrix and is expelled by centrifugation force or squeezing [133]. The amino acid composition and ionic strength play a major role in influencing WHC. A protein may bind water molecules to its charged groups, hydroxyl groups, peptide backbone groups, amide groups, and nonpolar residues of aminoacid, in which each group shows a varying capacity. Highly charged proteins have a stronger electrostatic attraction to water [12]. While salt improves the WHC at low ionic strength, it does not affect the hydration shell of proteins because the hydrated ions interact with the charged groups of proteins. In fact, as the concentration of salt increases, ions dehydrate the proteins by binding the existing water with themselves [134]. In some studies, the WHC of PPI obtained by the AE/IP method was 2.7 *g*/*g,* which is lower than soy protein [13]. Another study by Zhao et al. [135] reported that WHC values of commercially produced pea protein (3.38 *g*/*g*) are lower than soy protein (5.16 *g*/*g*). The water-binding ability of proteins is useful in food products such as sausages, puddings, doughs, and others where there is not enough water to dissolve protein. Still, it is hydrated by protein, which provides structure (swelling, gelation) and viscosity to the food [13].

### 5.3. Oil Holding Capacity

Oil holding capacity (OHC) or oil absorption capacity is defined as the amount of oil that can be absorbed per gram of protein. OHC values can be affected by a protein’s matrix structure, surface hydrophobicity, the type of lipid present, and the distribution and stability of lipids. Lipids and proteins interact through the binding of the aliphatic lipid chains to the non-polar side chains of amino acids; hence proteins with higher hydrophobicity tend to hold oils more strongly [12]. OHC increases mainly due to the physical entrapment of fat due to the hydrophobicity of protein. It is important to understand the factors that affect OHC to maintain product quality [136]. The OHC values for legume isolates are affected by the type and variety of legumes and the processing conditions used to produce the isolate. PPI obtained by the alkali extraction/isoelectric precipitation (AE/IP) method had an OHC value of 2.8 *g*/*g*, which was lower than SPI. Therefore, it was concluded that different extraction methods could significantly affect the OHC of PPI [13,133].

### 5.4. Emulsion Ability

Emulsifying ability is a term used to describe the ability of a compound to form emulsions composed of two liquids with different solubilities [109]. Proteins play an important role in emulsion formation and stabilization due to their amphiphilic nature and film-forming abilities [137]. Balanced hydrophobicity and hydrophilicity of proteins are required for good adsorption at the interface between the aqueous and oil phases [39]. Adsorption of proteins at the interface usually occurs in two stages. First, the proteins (including globulins and albumins) migrate and attach to the oil/water interface [74]. Due to its hydrophilic nature, protein can migrate to the interface depending on solubility [125]. Once the protein molecules are transferred and bound to the interface, the hydrophobic spots on the protein surface promote adsorption. During the second phase, structural rearrangement of proteins occurs, allowing protein molecules to partially denature and rearrange themselves so that hydrophilic parts face the aqueous phase. In contrast, hydrophobic parts remain in the oil phase [60]. This results in forming protein molecules in a viscoelastic film at the interfacial layer to stabilize the oil droplets. In an emulsion matrix, the adsorption of proteins at the oil/water interface is slow compared to low molecular weight emulsifiers, forming compact layers around oil droplets [138]. The emulsifying ability of pea proteins can be influenced by several factors, including protein concentration, protein structure, homogenization temperature/pressure, viscosity, pH, and protein-oil-water contact time [74]. The albumin fraction shows the best foaming and emulsifying properties because it preferentially accumulates at the oil/water interface. But low molecular weight hydrophilic albumins did not contribute too much to the emulsifying function. While in the globulin fraction, vicilin produces more stable emulsions, and legume has greater emulsion capacities. From this observation, it can be concluded that the vicilin fraction plays an important role in the emulsifying property of pea protein isolate [60]. As a function of pH values (3.0–9.0), pea protein had the lowest emulsifying capacity at pH values close to its isoelectric point (around pH 5). At pH values above 7, the emulsifying capacity is improved [74]. In addition, below pH 3 suggests that pea proteins show emulsification under acidic conditions than at neutral or alkaline pH [139,140]. At acidic pH, pea protein stabilizes emulsions with a gel-like network structure or acts as a Pickering stabilizer. Pickering emulsions can be characterized by solid particles adsorbing at the interface due to the partial double wettability of the oil and water interface [61]. Common methods used to form solid particles include heating, sonication, anti-solvent precipitation, and pH adjustment [140,141]. Emulsifying properties can be measured using various methods, including turbidimetric, droplet size measurement, and conductivity. The two common turbidimetric methods for measuring emulsifying properties are the emulsifying activity index (EAI) and the emulsifying stability index (ESI) [136]. In general, the application of pea protein as an emulsifier compared to soy protein isolates is still limited [39]. Some studies show that pea protein emulsion properties can be improved by heat treatment, high hydrostatic pressure, and pH treatment [83]. Ultra-high temperature effectively improves emulsion properties when pea protein concentrates have been subjected to micro-fluidization [75,142]. Emulsion properties have also been improved, creating a complex with various polysaccharides [28,143].

### 5.5. Gelation

The gel is a dispersed system with at least two components in which the dispersing agent can form a cohesive network. It is characterized by a lack of fluidity and elastic deformability [24]. Protein gelation is a process in which protein molecules embedded within an aqueous solvent form a three-dimensional network of molecular structures [144]. Gelation is one of the most important functional properties of globular proteins as it is used to change the texture of food [12]. The mechanism of globular protein gelation is a complex process consisting of multiple steps, including partial denaturation of protein molecules, gradual association or aggregation, and network formation. Protein gel can generally be divided into heat-induced and cold-set gelation of a three-dimensional matrix structure enclosing water, fat, and other food components. When the protein is heated above the denaturation temperature, the protein has a higher concentration than its lowest gelation concentration (LGC), resulting in partial expansion of the protein and exposing the interaction site, resulting in intermolecular interactions, eventually leading to the accumulation of protein aggregates to form a spatial gel network. The gel formed is termed heat-induced gelation [13]. Cold gelation of pea protein is a two-step process in which aggregates are formed by heating a low-concentration protein solution (<10%) to a pH far from its isoelectric point and without salts; and upon cooling, these aggregates combine to form a structured network by reducing electrostatic repulsions [28]. Pea protein extracted by ultrafiltration and diafiltration processes promotes the utility of aggregates as building blocks for cold-set gels [145]. The denaturation temperature of pea protein increased from 69 °C to 77 °C with increasing legume content. In comparison, the disulfide-bonded acidic and basic legume subunits were denatured and aggregated over a temperature range of 75 °C to 85 °C. During heat treatment, the detachment of legume oligomers and their rearrangements through hydrophobic interactions and exchange reactions of sulfhydryl/disulfide bonds occur simultaneously [7]. Most food protein gels are formed by heat treatment. Heat-induced/thermal gelation of pea proteins is studied by several researchers and reported to be influenced by many factors such as cultivars, extraction methods, protein heterogeneity, solvent parameters, and heating methods [146]. Recent studies have reported on the effect of transglutaminase on the gelation of pea protein fractions [71]. Unlike albumin, globulin (native or denatured) gives itself well to enzymatic gelation. Several studies have focused on heat-induced gelation of micellar casein suspensions in combination with pea protein isolates or pea protein fractions (Vicilin 7 S or Legumin 11 S concentrated fractions) [147].

In acid-induced cold gelation, the degree of aggregation of soluble pea protein further influences the strength of the acid gel. Compared to soluble and non-covalent vicilin thermo-aggregates, legume thermo-aggregates show reduced solubility and impair acidic gelation properties [148]. Salt-extracted PPI leads to the formation of a gel network, which was evaluated by dynamic rheological measurements, indicating that the gel point was dependent on the heating rate but was not affected by the cooling rate [7]. In a comparative study, the ideal conditions for the formation of strong heat-induced gels of pea protein were 19.6% (*w*/*w*) protein content, pH 7, and heating up to 93 °C. The gels made with soy protein isolates under the same conditions were stronger and more elastic than those made with pea protein [61].

### 5.6. Foaming Properties

Foaming capacity and foaming stability are the two terms used to describe the foaming properties of the protein [13]. Forming capacity is defined as the amount of interfacial area produced by protein. It is associated with the average hydrophobicity of proteins, and partial denaturation is increased by surface activity. The ability of the protein to stabilize a foam against stresses is referred to as foaming stability. The stable protein-based foam has cohesive interfacial films due to hydrogen bonding and electrostatic and hydrophobic reactions [12]. In terms of pea protein, the ability to form foam is dependent on several factors like pH, protein concentration, ionic strength, viscosity, temperature, and extraction method. Pea protein concentrates were found to form more stable foam than isolates due to a higher concentration of polysaccharides [149]. The highest foaming capacity of pea protein at pH 3 with a maximum value of 81% and lower at pH 5 indicate that protein aggregation at the isoelectric point had a negative effect on the ability to entrap and unfold air particles. At pH 3, protein gains net charges; the higher FC values may be due to a higher level of protein unfolding. However, FS is also influenced by net protein charge, which limits interaction between entrapped air particles [83]. Some studies found that pea protein isolates extracted by salt extraction give better foaming properties than alkaline extraction. High-pressure modification treatment enhances FC up to 19–35% but depends on pH and protein concentration [150]. Protein unfolding by high-intensity ultrasound treatment increases surface hydrophobicity and reduction in particle size, thereby promoting the adsorption dynamics at the air-water interface and, as a result, increasing the foaming capacity of pea protein [151].

## 6. Pea Protein Application and Its Health Benefits

### 6.1. Food Emulsifier

Pea protein provides comparable emulsifying properties that allow it to meet current consumer demands for alternative plant-based protein sources. The emulsifying properties depend upon the pea’s cultivar, the isolated protein’s composition, structure, and physicochemical properties, which may be affected by the isolation methods and conditions [60]. Pea protein has been used as an emulsifier in liquid emulsions and spray-dried emulsions for the microencapsulation of oil [152]. The inconsistent behavior of pea protein is due to its limited molecular flexibility, which prevents it from making a stable interfacial film when more oil is present. Pea protein form a rigid membrane at the oil-water interface, reduces the interfacial tension between water and oil and stabilize emulsions. It has high surface-active characteristics at the oil-water interface [142]. The ability of the protein to form stable foams is a significant property in cakes, muffins, whipped toppings, fudges, etc. [83]. According to some studies, pea protein is a better emulsifying and foaming agent than soy protein at neutral pH [7].

### 6.2. Encapsulation Techniques for Bioactive Ingredients

Bioactive compounds have health benefits, including antioxidant, anticancer, and anti-inflammatory properties. However, they are sensitive to pH, light, and thermal treatments, as well as their hydrophobic or crystalline nature with low water solubility may limit their utilization in food products [153]. To solve this problem, encapsulation is a promising technique [13]. Nowadays, pea protein is used as an encapsulation material due to its health benefits, hypoallergenic aspects, and no genetic modification aspects [154]. The three main technologies involved in using pea proteins as encapsulation materials are spray drying, emulsion, and complexes. In pea protein-based encapsulation systems, spray drying is a commonly used encapsulation technique to produce microparticles. Many lipophilic bioactive ingredients, such as β-tocopherol, omega-3 fatty acids, linolenic acid, conjugated linoleic acid, and black pepper oil, were first encapsulated in feed emulsions and stabilized by pea protein and then obtained into microparticles by spray drying [24,155,156]. Comparing freeze drying to spray drying, the freeze-drying encapsulation technique is more suitable for heat-sensitive materials containing bioactive ingredients. The docosahexaenoic acid (DHA) capsules with different wall materials are produced by freeze-drying. Both PPI and PPI-modified starch complexes based on microencapsulation provide good protection for DHA against oxidation during storage [60]. Riboflavin is encapsulated in pea protein microparticles cross-linked by transglutaminase, and the release properties of this system were studied in simulated gastric and intestinal fluids. Depending on the rate of the loaded core, the encapsulation efficiency of crystallized riboflavin varied from 74% to 84% [123].

### 6.3. Pea Protein-Based in Films

Pea protein as a biocompatible and biodegradable natural polymer has been extensively studied for the production of edible/biodegradable films. It offers a promising possibility for applying pea protein-based films in the food industry [13]. Pea protein-based film forms reference objects, processing conditions, and parameters. In general, the desired film has excellent gas and water barrier properties (e.g., low oxygen permeability and low water vapor permeability and excellent mechanical properties such as tensile strength, modulus, puncture resistance, and good appearance properties). The film-forming properties of pea protein isolate are influenced by the type of plasticizer, protein-plasticizer ratio, pH, heat treatment, and injection parameters [157]. Combining pea protein concentrates with glycerol results in greater homogeneity of surface structure and lower light transmission in films compared to films based on whey proteins. At the same time, their physical and mechanical properties were comparable [158]. Some other studies showed that mixing pea protein with sorbitol form films with good tensile strength and transparency [159]. A combined formulation of acetylated cassava starch and pea protein isolates improves film formability and mechanical properties. In particular, pea protein isolates have increased film stability, tensile strength, protein aggregation, surface hydrophobicity, and barrier properties to water vapor and oxygen [160].

### 6.4. Health Attributes of Pea Proteins

Over the past decade, there has been an increased demand for plant-based proteins. It reduces the risk of obesity, high blood pressure, and diabetes. For most plant-based proteins, at least one essential amino acid is absent, but pea protein is rich in lysine, which helps to maintain a healthy immune system [161]. Hypertension is directly associated with the development of cardiovascular disease in humans. Antihypertensive peptides from pea protein were usually characterized as inhibitors of angiotensin I-converting enzyme, given the essential role of the renin-angiotensin system in regulating BP [13]. Another study reported that angiotensin-converting enzyme2 from pea protein is considered a strategy for identifying antihypertensive capacity [14]. The potential benefits of consuming pea protein result in lowering satiety, food intake, and blood glucose [8]. Pea protein suppressed postprandial glycemia and reduced pre-and post-meal blood glucose in humans [162]. Satiety, a feeling of fullness, has an immeasurable impact on weight control due to its ability to affect the body’s caloric intake [163]. PPIs’ satiety properties were given in the first study in vitro gastric conditions for the digestion of pea, whey, and casein proteins. Unlike whey, pea protein transiently aggregates during gastric digestion but has much less precipitation than casein, allowing for intermittent gastric retention and contributing to a satisfactory effect. In addition to adequate training, pea protein supplementation promoted greater increases in muscle strength [164]. Dyslipidemia is also an important risk factor for cardiovascular disease. Proper management of diet and blood cholesterol reduces the risk of heart disease. Pea protein can be part of the precautions. A pea diet resulted in lower total cholesterol, lower lipoproteins, and reduced caloric intake. Multiple studies have shown that when combined with oat and wheat fiber, pea protein results in significant reductions in total cholesterol [162].

### 6.5. Commercially Available Pea Protein Products

**Figure 7:**
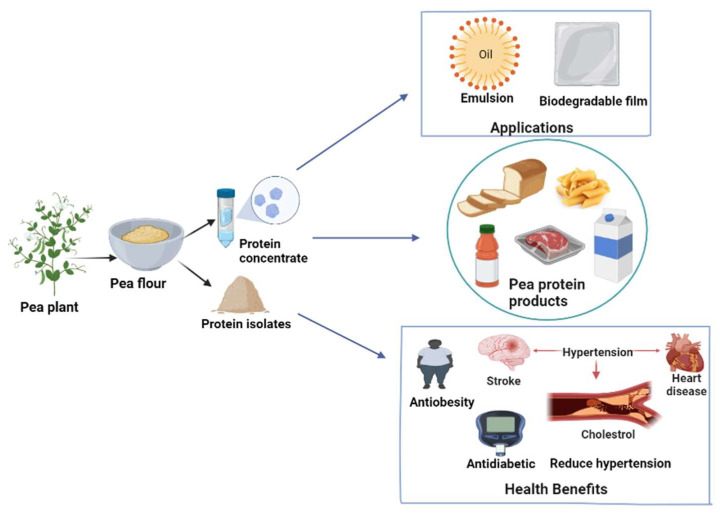
A schematic representation of pea protein applications and their benefits.

Pea proteins are considered functional ingredients to enhance the protein content in the diet while providing some functionality (gelling and thickening agents, stabilizers of emulsions and foams, acting as binding agents for fat). They also have biological activities such as antioxidant or antimicrobial characteristics [104,153]. For human consumption, pea protein can be used in cereal, bakery, meat, and dairy products. This section depicts the area in which pea protein has been extensively used, as given in Figure 7.

#### 6.5.1. Cereal and Bakery Products

##### Bread

Adding pea protein to cereal products helps improve the products’ nutritional properties by providing them with essential amino acid profiles and improves texture and structure [35,65]. Commercial pea protein products are mainly in concentrated forms with less than 85% of protein content (dry weight basis) and do not contain any gluten, so it is used for the production of gluten-free foods [7]. The nutritional quality of wheat protein has been improved by substituting 20% of wheat flour with pea protein (85% protein), which results in the dough gluten network becoming weak and reducing bread volume leading to the compact crumb structure (small crumb cells) with hard texture [165]. Generally, gluten-free bread is made with high content of starchy ingredients; consequently, increasing proteins in such formulations will ensure a better nutritional composition. Pea proteins have the potential to nutritionally enrich this type of food and also contribute to protein networks. When using a high content of proteins, hydration of water is adjusted due to the high-water holding capacity, which reduces the impact on crumb hardness [166]. Bread made by adding maize starch and pea proteins (70:30) had slow digestible starch [167].

##### Pasta

The protein is used in pasta and noodle applications, in traditional durum wheat noodles, and Chinese vermicelli pea protein is present. Pasta is traditionally made from durum wheat semolina and is based on a low temperature, low shear extrusion process followed by drying [8]. In pasta production, pea protein is used to fortify the nutrition of the pasta and combined with many ingredients to vary the amount of addition up to 12.5%. For example, eggless pasta of acceptable hardness is formulated with a combination of pea protein (84–88% protein), extruded and non-extruded quinoa flour (red and white), potato starch, and tara gum [168]. The lower water absorption of pasta fortified with pea protein can be a factor in determining the high hardness of cooked pasta. The denatured pea protein did not affect the texture or sensory properties of the noodles, but in vitro studies show reduced glucose release [169]. This is associated with a stronger interaction between protein and starch, reducing the gelatinization degree. Pea protein interacts with starch, which limits the gelatinization process, but these interactions depend on the structure of the pea protein. The interaction of hydrolyzed pea protein with corn or cassava starch reduces the apparent viscosity of the paste during heating and cooling, weakening the starchy gel. The lack of a strong covalent protein network in 100% pea protein pasta results in an overall weak pasta structure that facilitates water penetration during cooking [28].

#### 6.5.2. Extruded Snacks

The extrusion technology can be categorized into two low moisture extrusion (less than 35%) and high-moisture extrusion (greater than 40%); both are widely used in commercial food production. In general, LME is used in preparing extruded snacks, while HME is used in preparing meat analogs [18,170]. Pea ingredients can be used for advanced snacks and breakfast cereals. Extrusion cooking is a continuous process where starch and protein are plasticized and cooked with moisture, pressure, temperature, and mechanical shearing. In extrusion cooking, the starch is gelatinized, the protein is denatured, and it is inactivated by 90% more anti-nutritional components [8]. Pea protein isolate fortified extruded products have high protein content and a balanced amino acid profile compared to pure starch extrusion. In addition, PPI-enhanced extrusions with high nutritional value and desirable physicochemical properties can be created by controlling the protein content and parameters of the extrusion process [13,171].

#### 6.5.3. Beverages

Fortification of beverages includes adding micronutrients to different beverages consumed by different consumers. Pea protein is used in fortified beverages like protein shakes, sports drinks, and protein juice mixes [172]. When developing beverages fortified with pea protein components, the most crucial functional properties are protein solubility, thermal stability, and rheological behavior. Protein drinks require heat treatment such as UHT (Ultra High-Temperature Treatment) for safety and stable shelf life. Currently, to avoid astringency sensorial defects, protein drinks are ideally prescribed near pH 4–6 [76]. Pea protein has a net negative charge at neutral pH and repels each other in solution. During the acidification process, pea protein loses its net negative charge and becomes neutral, resulting in the weakest hydration around the isoelectric point (pH value around 4.8). As a result, when pea protein products are acidified and heated, they quickly aggregate and sediment in the final products [173]. Pea protein-based beverages have a stronger flavor that may be associated with lipid oxidation during heat treatment and the release of compounds resulting from the Maillard pathway [174]. The potential factors influencing the formation of soluble complexes in pea protein-polysaccharide systems are most important to developing desirable pea protein fortified beverages [28].

#### 6.5.4. Dairy Products

The first vegetable protein used in dairy products is soy protein because they are readily available and of good quality. However, increasing concern about allergens, genetically modified organisms (GMOs) free, and phytoestrogens have highlighted the relevance of pea protein in the marketplace. Pea protein has established itself in special nutrition with very good digestibility and an almost complete amino acid profile. Taking sports nutrition products as a pea protein because of its high content of three essential branched-chain amino acids (BCAAs), leucine, isoleucine, and valine, promotes muscle growth [7,168,175].

#### 6.5.5. Meat Products

The peas ingredients have been used in various forms of meat and meat analog applications, depending on the formulation, technology, and regulatory compliance. The pea protein bind water and fat and create a firm texture due to the amylose content, starch retrogradation, and gel formation. These properties make it unique and effective as binders, fillers, and functional enhancers. Processed meat products are traditionally fortified with a wide range of ingredients (proteins, spices, starches, etc.) due to their functionality, taste, and texture properties. Pea protein can impair the properties of foods, but it exhibits excellent properties for producing processed meat products. Adding PPI to ground meat patties has to produce softer beef patties, tender, require less compression than pure beef patties, and retain more fat than regular beef patties [65,83]. In cooked restructured steaks, adding pea protein isolate (8%), besides enhancing the protein content, increases its hardness, chewiness, cohesiveness, and gumminess due to the ability of the pea protein to bind water and fat as well as gelling property. It becomes better when combined with a transglutaminase uniform structure [176]. Chicken nuggets were enriched with pea protein isolate (83% protein) at a 12% level, increasing the protein content up to 39% when compared to the control (35%), but pH and ash contents were not affected. In these products, pea protein reduces cooking losses due to the high binding capacity of the pea protein, which leads to a stronger network. However, when high amounts (greater than 9%) of pea protein were used, sensory problems related to green notes were observed in pea protein-enriched nuggets [177,178]. Therefore, some more studies are needed to reduce the odor of beans.

## 7. Conclusions and Future Perspective

Over the past few years, the utilization of pea protein has been a growing trend in the global food industry due to its diverse industrial application, including its emulsifying, gelling, binding, and film-forming ability. In keeping with emerging consumer trends, the food industry is searching for protein ingredients to replace other plant and animal-based proteins. Pea protein has gained much interest due to its availability, cost-effectiveness, allergenicity, and high nutritional value. In addition, it has excellent functional qualities, is gluten-free, and is a nongenetically modified organism. Furthermore, pea protein is an excellent ingredient for improving the nutritional and technological properties of cereal and bakery products, meat and dairy products, and beverages. However, like other plant proteins, pea protein as a food ingredient has some limitations in terms of its functionality, flavor, and color. Studies have reported that pea protein may form weaker and less elastic gels than soybean protein during food processing. Thus, more research should be conducted to improve protein functionality, reduce undesirable color and flavor compounds, and improve the process to limit the damage to protein. Moreover, public education is needed to promote the acceptance of pea protein as a healthy food. By raising consumer awareness of its health benefits, it can be expected that the global market will extensively use pea protein in food ingredients, beverages, sports supplements, bakery products, meat products, and dairy products.

## Figures and Tables

**Table 1:** Various extraction methods, yield, and application.

Extraction Method	Protein Yield (%)	Application	References
Alkali extraction/isoelectric precipitation	62.6–80	Improve texture and nutritional quality of food. Emulsifying material	[54]
Dry fractionation	50–77	Used encapsulating material	[55,56]
Salt extraction	68.2–74.8	Foaming capacity increases	[11,57]
Mild fractionation	55–65	Used to produce biodegradable natural polymer	[51,55,58]

**Table 2:** Amino acid profiling of pea protein in g/100g.

Amino Acid	Pea Protein (g/100 g)	References
Essential amino acid		[63,64,65]
Valine	2.7–5	
Leucine	5.7–6.4	
Isoleucine	2.3–4.5	
Methionine	0.3–1.1	
Phenylalanine	3.7–5.5	
Tryptophan	0.7–1	
Threonine	2.5–3.9	
Lysine	4.7–5.7	
Histidine	1.6–2.5	
Non-essential amino acid		[5,66,67]
Alanine	3.2–4.3	
Aspartic acid	8.9–11.5	
Cystine	0.2–1	
Glutamic acid	12.9–13.2	
Glycine	2.8–4.1	
Proline	3.1–4.5	
Serine	3.6–5.3	
Tyrosine	2.6–3.8	

**Table 3:** Classification of pea protein and its molecular characteristics.

Protein	Content	Solubility	Molecular Weight	Distinct Structural Features	Reference
Globulin	65–80%	Salt solution			
Legumin			320–400 kDa	Hexameric protein with six subunits. Compact Quaternary structure. Has an acidic and basic polypeptide linked by disulfide bonds	[7,78]
Vicilin			150–170 kDa	Trimeric protein. Combination of heterogenous polypeptides with no disulfide protein. Has hydrophilic surface more than legumin	[12,28]
Convicilin			180–210 kDa	It can form trimers, including N-terminal with three convicilin molecules. Contain sulfur-containing amino acid.	[60]
Albumin	10–20%	Water solution	5–80 kDa	Two major fractions: a larger albumin protein comprising two polypeptides and a minor one.	[54]
PA1	5–9%		10 kDa	Dimer	[28]
PA2	10–20%		50 kDa	Dimer	[12]
Lectins	2.5%		50 kDa	Tetramer	[16]
Lipoxygenases	<1%		n/a	n/a	[54]
Serine/trypsin protease inhibitors	<2%		10–16 kDa	Monomer	[7]
Prolamin	4–5%	Alcohol solution	n/a	Present in a small amount. Has high glutamine and proline content.	[37]
Glutelin	3–4%	Insoluble	n/a	Class of prolamin-like protein. Only soluble in dilute acid or bases. Rich in hydrophobic amino acids.	[79]

**Table 4:** Different modification methods and their characteristics.

Physical Modification	Modified Characteristics	Reference
High-pressure treatment (HPP)	Structural changes, foaming stability, and emulsifying property enhanced	[82]
Heat with shear treatment (Extrusion)	Improve the texture of protein	[122]
Cold atmospheric pressure plasma treatment	Improve solubility, emulsifying ability, and water holding capacity	[99]
Ultrasonic treatment	Improve gelling properties and enhance solubility	[101,102]
**Chemical Modification**		
Glycation	Helps to reduce beany flavor	[103,123]
Acylation	Helps to improve solubility, Emulsion stability, water holding capacity, and foaming properties.	[116]
Deamidation	Improve solubilityReduces unpleasant beany flavor, bitterness, and lumpiness	[80]
**Biological Modification**		
Fermentation	Improves digestibility of protein	[13]
Enzymatic modification	Improves protein solubility, hydrophobicity, emulsifying and foaming properties	[124]

## Data Availability

Data sharing is not applicable to this article.
